# Efficacy of N-Acetylcysteine Augmentation on Obsessive Compulsive Disorder: A Multicenter Randomized Double Blind Placebo Controlled Clinical Trial

**Published:** 2017-04

**Authors:** Ahmad Ghanizadeh, Mohammad Reza Mohammadi, Shahla Bahraini, Zahra Keshavarzi, Ali Firoozabadi, Ali Alavi Shoshtari

**Affiliations:** 1 Research Center for Psychiatry and Behavioral Sciences, Shiraz University of Medical Sciences, Shiraz, Iran.; 2 Department of Psychiatry, School of Medicine, Shiraz University of Medical Sciences, Shiraz, Iran.; 3 Psychiatry and Psychology Research Center, Roozbeh Hospital, Tehran University of Medical Sciences, Tehran, Iran.; 4 Department of Psychiatry, Roozbeh Hospital, Tehran University of Medical Sciences, Tehran, Iran.

**Keywords:** *N-Acetylcysteine*, *Adjuvant*, *Obsessive Compulsive Disorder*, *Treatment*, *Clinical Trial*, *Placebo*

## Abstract

**Objective:** Glutamate is considered a target for treating obsessive-compulsive disorder (OCD). The efficacy and safety of the nutritional supplement of N-Acetylcysteine (NAC) as an adjuvant to serotonin reuptake inhibitor (SSRI) for treating children and adolescents with OCD has never been examined.

**Method:** This was a 10-week randomized double-blind placebo-controlled clinical trial with 34 OCD outpatients. The patients received citalopram plus NAC or placebo. Yale-Brown Obsessive-Compulsive Scale (YBOCS) and Pediatric Quality of Life Inventory (PedsQL™) were used. Adverse effects were monitored.

**Results:** YBOCS score was not different between the two groups at baseline, but the score was different between the two groups at the end of this trial (P<0.02). The YBOCS score of NAC group significantly decreased from 21.0(8.2) to 11.3(5.7) during this study. However, no statistically significant decrease of YBOCS was found in the placebo group. The Cohen’s d effect size was 0.83.

The mean change of score of resistance/control to obsessions in the NAC and placebo groups was 1.8(2.3) and 0.8(2.1), respectively (P = 0.2). However, the mean score of change for resistance/control to compulsion in the NAC and placebo groups was 2.3(1.8) and 0.9(2.3), respectively. Cohen’s d effect size was 0.42.

The score of three domains of quality of life significantly decreased in N-Acetylcysteine group during this trial. However, no statistically significant decrease was detected in the placebo group. No serious adverse effect was found in the two groups.

**Conclusion:** This trial suggests that NAC adds to the effect of citalopram in improving resistance/control to compulsions in OCD children and adolescents. In addition, it is well tolerated.

Obsessive-compulsive disorder is a neuropsychiatric disorder with the prevalence of 1.8% among school aged (1). Estimated OCD heritability in children ranges from 45% to 65% (2). 

Serotonin specific reuptake inhibitors (SSRI) are commonly administered for treating children and adolescents with OCD (3).

It seems that SSRIs are better tolerated than clomipramine (4). However, resistance to treatment is common (5). Only 40-60% of patients with OCD respond to SSRIs. In other words, about 40 to 60% of patients with OCD do not completely respond to SSRIs (5). In these cases, antipsychotic augmentation strategy is usually advised. However, only one-third of medication-resistance OCD patients show a meaningful response to this intervention (6).

Therefore, not only its effect is limited to a minority of patients, but also there are some concerns about long-term use of antipsychotics (7).

It is proposed that glutamate plays a significant role in pathophysiology of pediatric obsessive-compulsive disorder (8). Considering the glutamate role, novel therapeutic strategies are suggested. N-Acetylcysteine (NAC) which is an amino acid derivative has antioxidant effects. NAC, which is a health supplement, targets glutamatergic system (9).

NAC is converted to cysteine, which is a substrate for the glutamate/cystine antiporter and increases extracellular glutamate (10). The increased glutamate reduces the synaptic release of glutamate (11). NAC decreases oxidative stress through its neuroprotective role (12). NAC has been tested for treating some symptoms of autism (13, 14), repetitve behavior of nail biting (15), cannabis-dependence in adolescents (16), and trichotillomania (17).

NAC is suggested for treating OCD (18). A case report indicated that NAC augmentation of fluvoxamine significantly decreased YBOCS score in a patient with OCD symptoms (19). A retrospective chart review of six treatment resistant OCD patients, who had been treated with NAC (2833.3±408.2 mg/day) for 6–12 weeks, reported that only one patient responded to NAC. Moreover, the severity of symptoms was exacerbated in two patients. Meanwhile, no serious adverse effect was reported (20). 

Only one published randomized double blind controlled clincal trial investigated NAC augmenting with SSRIs in adult patients with OCD (21). N-acetylcysteine (up to 2400 mg/d) decreased YBOCS score more than placebo. In a study, 52.6% of patients in the N-acetylcysteine group showed full response while the rate in the placebo group was 15% (P = 0.013) (21). However, there is a debate that children with OCD are not little adults with OCD (22). Children with OCD more than adults have simple tic-like compulsions (22). OCD is very heritable in children and adolescents (2). There is a negative relationship between insight and OCD severity in children (23), and poor insight is associated with poorer outcome (23). Improving insight or resistance to compulsion increases the rate of successful cognitive behavior therapy in children (23).

The quality of life of adolescents with OCD is less than that of those without OCD (24). In addition, health related quality of life is associated with responders and non-responders or relapsers and non-relapsers in OCD, suggesting an association between the OCD symptoms and quality of life (25). No published study has ever examined the effect of NAC on the quality of life of patients with OCD. 

This trial assesses the potential efficacy and safety of augmentation of treatment with serotonin reuptake inhibitors (SSRIs) with NAC. In addition, we examined the effect of NAC on the quality of life of patients. Considering the previous case reports and non-controlled trials in children and adolescents with obsessive-compulsive disorder, it is hypothesized that this adjuvant therapy is effective for treating children and adolescents with OCD.

## Materials and Methods

This was a multi-center ten-week randomized placebo-controlled double-blind parallel-group trial performed in the outpatient clinics of Shiraz University of Medical Sciences and Roozbeh Psychiatric Hospital (affiliated to Tehran University of Medical Sciences, Tehran, Iran) from March 2011 to April 2012.

The age range of patients was 10 to 21 years from both genders. The patients and at least one of their parents were interviewed according to DSM-IV-TR diagnostic criteria and Kiddie Schedule for Affective Disorders and Schizophrenia (K-SADS). They met the diagnostic criteria for OCD, and their OCD symptoms chronicity was at least one year.

Those with substance use, serious uncontrolled medical conditions such as thyroid dysfunction, clinically estimated mental retardation, primary diagnosis of psychosis, suicidal thought, current Tourette disorder or ADHD needing treatment, pregnancy, breast feeding, lack of adequate and reliable contraception, receiving concurrent psychotherapy, and patients who were unable to understand or follow instructions were excluded. 

The Ethics Committee of Shiraz University of Medical Sciences approved this study and it was performed according to the Declaration of Helsinki ¬and its later revisions. The patients/caregivers provided written informed consent before entering into the study. 

Thirty-four outpatients with obsessive-compulsive disorder were randomized into one of the two groups. A computerized random number generator was used for randomization of these patients. The patients and the researchers who rated the patients were blind to the group allocation. The number of patients in the NAC group was 19 and the number in the placebo group was 15.

In this 10-week trial, one group received citalopram (20 to 40 mg/day) and N-Acetylcystein (titrated up to 2400mg/day), and the other group received citalopram (20 to 40 mg/day) plus placebo. One patient in the NAC group received Fluoxetine during this trial (20 mg/day). Two other patients in NAC received Fluvoxamine (50 mg/day) or Serteralin (100 mg/day). One patient in the placebo group received Serteralin (100 mg/day). The patients did not receive any serotonin reuptake inhibitors with adequate therapeutic duration and dosage in the last one month before randomization. The patients failed to respond to at least a previous a trial of an SSRI. The dose of NAC during the first week of the trial was 600 mg/day, which increased to 1200 mg/day in two divided doses in the second week. The patients were administered 1800 mg/day during weeks 4 and 5. The daily dose for NAC from week 6 to the end of this trial was 2400 mg/day. The dose could be adjusted considering clinical symptoms. The maximum dose for six patients was 1200 mg/day, and 2400 mg/day was administered for the rest of the patients. 

Both NAC and placebo were in the form of identical tablets. No significant change of concurrent medication was allowed during the trial. Six patients dropped out from this trial. All of these dropped outs withdrew during the first month of the intervention. Primary outcome measure was assessed at baseline, weeks 4, 8, and 10. Adverse effects were assessed at weeks 2, 4, 8, and 10. The patients and their caregivers were provided a phone number to be able to contact one of the researchers whenever they had any question or concern about this trial or experiencing any adverse effect. Moreover, the patients were advised to refer to the emergency psychiatry department in case of any emergency adverse effects.

The main outcome was assessed using Yale Brown Obsessive Compulsive Scale (Y-BOCS) (26), which is a 10-item semi-structured questionnaire measuring the severity of obsessive- compulsive disorder symptoms during the last week. The changes of the total score of Y-BOCS were examined during this trial.

The scores of items 4 and 5 of the YBOCS including “resistance against obsession” and “degree of control over obsessive thoughts” were summed up representing resistance/control to obsessions. The scores of items 9 and 10 of YBOCS including “resistance against compulsions” and “degree of control over compulsive behaviors” were summed up to measure the resistance/control to compulsions. Lower scores represent higher resistance/control over symptoms (27). 

Quality of life was assessed using Pediatric Quality of life Inventory (PedsQL™ 4.0) Generic Core Scales (28, 29) . The effect of intervention on physical functioning, emotional functioning and social functioning was assessed. Physical functioning consists of eight questions. Emotional functioning and social functioning consists of 5 questions each. Each question ranges from 0 to 4 on a Likert scale. Higher scores show worse conditions. Some of the examples of the physical functioning are: “Hard to run” and “hard to sport or exercise”. The following two statements are examples of emotional functioning domain: “Feel sad or blue”, and “trouble sleeping”. Two example statements of the domain of social functioning are: “Trouble getting around with peers” and “teased”.

Adverse effects were recorded through a self-report checklist and a physical examination. 

***Statistical Analysis***

A previous trial showed that NAC decreased YBOCS more than placebo. The mean (SD) difference between the two groups was 5.14 (2.5) (21). Assuming significance level of 5% and a power of 95%, a sample size of 14 was calculated. 

Statistical analysis was performed using the Statistical Package for Social Sciences, Version 11.5 (SPSS 11.5) for windows. Gender ratio was compared between the two groups using chi-squared test. The mean of age was compared between the two groups using the non-parametric test of the Mann–Whitney U Test. Intent-to-treat, and the last observation carried forward, were performed to handle the missing data. Wilcoxon Signed Ranks Tests were performed to examine the changes of YBOCS score within groups. 

The mean difference of scores at baseline and the end of the trials were compared between the two groups. The p value for Kolmogorov-Smirnov test was 0.9 concerning YBOCS score. However, since non-parametric tests are more conservative, they were run. 

The change of Peds quality of life score was compared between the two groups using the non-parametric tests of Mann–Whitney U.

## Results

**Figure 1:**
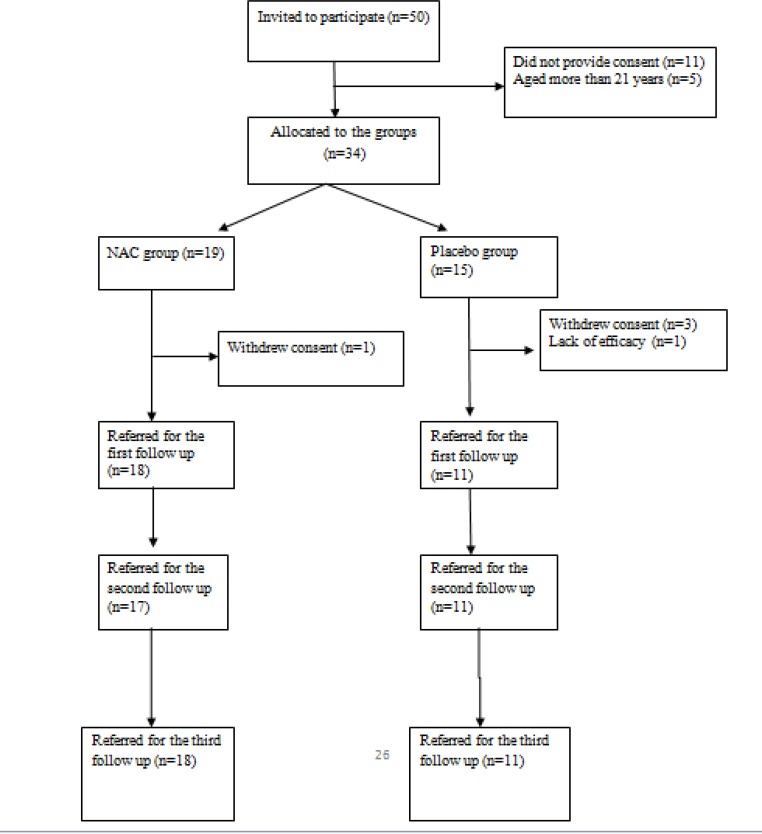
Flowchart for the Clinical Trial of N-Acetylcysteine (NAC) Group versus Placebo Group

Thirty-four patients were randomly allocated into one of the two groups. Five patients did not return for the first month follow up assessment (four patients withdrew their consent and one patient withdrew because the treatment was not effective). Twenty-nine out of 34 patients completed this trial (Figure 1). The demographic characteristics of the patients who were visited for at least one month after the randomization are displayed in Table 1. 

Gender ratio, the mean age, and YBOCS score at baseline were not statistically different between the NAC and placebo groups. The YBOCS score was different between the two groups at the end of this study (P<0.02). 

**Figure 2:**
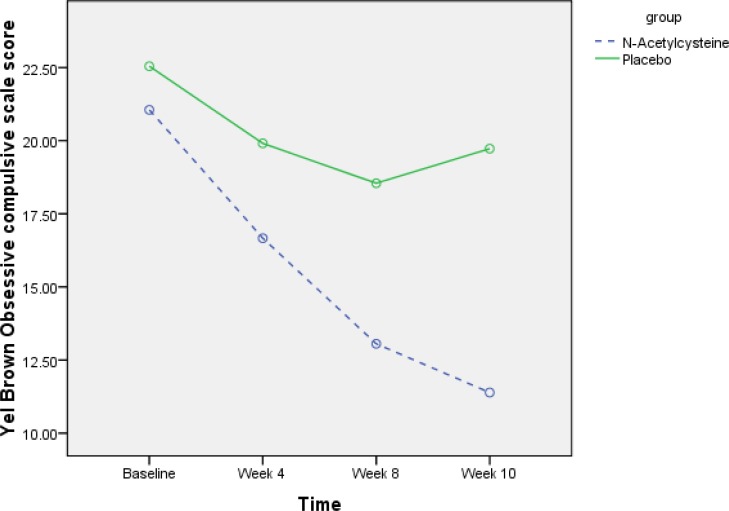
Changes of Yale Brown Obsessive Compulsive Scale Score in the Two Groups during

The YBOCS score decreased from 21.0(8.2) to 11.3(5.7) in the NAC group during this trial (Figure 2). The change of score in the placebo group was from 22.5(8.4) to 19.7(9.7). The Cohen’s d effect size was 0.83. Wilcoxon Signed Ranks Tests showed that the score of YBOCS significantly decreased in NAC group from the week 4. The difference of YBOCS at weeks 4, 8, and 10 was statistically less than the score at baseline in the NAC group. Meanwhile, the score at weeks 4, 8, and 10 were not statistically different from the YBOCS score at baseline in the placebo group (Table 2). Using Mann-Whitney U test, we compared the two groups with respect to the mean difference of score between weeks 4, 8, and 10 with the score at baseline. The mean difference was not statistically different between the two groups at weeks 4 and 8 (P = 0.3, and P = 0.1, respectively). However, there was a trend for statistically significant difference between the two groups in the mean difference f YBOC at week 10 (P = 0.06).

The mean change of score of resistance/control to obsessions in the NAC and placebo groups was 1.8(2.3) and 0.8(2.1), respectively. The Cohen’s d effect size was 0.42, with no between group difference (P = 0.2). However, the mean score of change for resistance/control to compulsion in the NAC and placebo groups was 2.3(1.8) and 0.9(2.3), respectively (t = 2.5, df = 32, P<0.04). The score decreased from 4.7(1.7) to 3.6(2.6) in the placebo group, while it decreased from 4.7(1.9) to 2.3(1.6) in the NAC group. 

The changes of different domains of quality of life scores showed that all the domains scores, except for physical function, decreased in both groups during this trial. 

**Table 1:** Age, Gender Ratio, and Yale-Brown Obsessive Compulsive Scale Score Mean During this Trial in Both Groups

**Variable**	**NAC group (n=18)**	**Placebo group (n=11)**	**Significance**
Mean (SD) years of age	16.5(2.9)	15.9(3.7)	P=0.6
Number (%) of female	7(38.9)	7(63.8)	P=0.1
YBOCS score at baseline	21.0(8.2)	22.5(8.4)	P=0.6
YBOCS score at week 4	16.6(6.9)	19.9(6.1)	P=0.2
YBOCS score at week 8	13.0(7.0)	18.5(7.9)	P=0.06
YBOCS score at week 10	11.3(5.7)	19.7(9.7)	P<0.02

Mann–Whitney U Tests were run except for gender ratio comparison which Chi-square tests was run.

**Table 2:** Within Group Changes of YBOCS Score in the Two Groups during This Study

	**Group**	**Mean(SD)**	**Significance**
**Difference between ** **baseline and week 4**	NAC	4.3(4.8)	0.001
	Placebo	2.6(9.7)	0.3
**Difference between ** **baseline and week 8**	NAC	8.0(5.9)	0.003
	Placebo	4.0(97.9)	0.3
**Difference between ** **baseline and week 10**	NAC	9.6(6.6)	0.001
	Placebo	2.8(9.7)	0.3

Wilcoxon Signed Ranks Tests were performed.

**Table 3:** The Within Groups Changes of Quality of Life Score during this Study

		**Baseline**	**10 weeks**	**Significance**
**Physical function **	NAC group	11.1(5.1)	6.8(5.2)	0.005
	Placebo group	10.4(7.0)	9.9(98.1)	0.9
**Emotional function**	NAC group	9.3(4.5)	4.2(4.2)	0.001
	Placebo group	17.0(11.5)	19.0(7.7)	0.02
**Social function **	NAC group	7.7(4.8)	3.3(3.7)	0.001
	Placebo group	7.7(5.6)	5.4(4.0)	0.049

Mann–Whitney U Tests were run.

**Table 4:** Number of Adverse Effects in the N-Acetylcysteine and Placebo Groups

**Adverse effect**	**NAC group**	**Placebo group**	**Significance**
Constipation	2	3	.3
Dizziness	6	3	1.0
Hypotension	1	2	.5
Dry mouth	4	4	.4
Blurred vision	5	2	.6
Sweat	10	2	.06
Urinary disturbance	2	1	1.0
Palpitation	2	1	1.0
Fatigue	11	4	.2
Tremor	5	1	.3
Anorexia	3	4	.3
Nervousness	4	3	1.0
Agitation	5	3	1.0
Headache	3	4	.3
Insomnia	5	2	.6
Anxiety	5	3	1.0
Diarrhea	2	0	.5
Nausea	1	1	1.0
Drowsiness	1	0	1.0
Rash	1	0	1.0
Cramp	2	1	1.0

The score of physical function in NAC group decreased from 11.5(5.1) to 6.8(5.2), (P<0.005). However, the score did not significantly decrease in the placebo group (P = 0.9) (Table 3). Moreover, the mean changes of the scores during this trial were not different between the two groups (Table 4).

The number of adverse effects in both groups of NAC and placebo are displayed in Table 4. The rate of none of the adverse effects was statistically different between the two groups. The most common adverse effects in NAC group were as follows: Fatigue (n =11), sweating (n = 10), dizziness (n = 6), blurred vision (n = 5), insomnia (n = 5), and tremor (n = 5). However, the most common adverse effects in placebo group were as follows: Fatigue (n = 4), anorexia (n = 4), headache (n = 4), and dry mouth (n = 4).

## Discussion

The main aim of this trial was to examine the efficacy of NAC augmenting for treating OCD in children and adolescents. To the best of our knowledge, this was the first double blind randomized placebo controlled clinical trial to examine the efficacy of NAC and SSRIs for treating children and adolescents with OCD. The only trial investigating the efficacy of NAC for treating OCD included adults (21). There is very limited current knowledge about the role of NAC in treating OCD. Our results are in similar line with those of the previous clinical trials and a case report about effectiveness of NAC (19, 21). One clinical trial showed that NAC (2400 mg/day) decreased 10.87(2.94) of the score of YBOCS during 12 weeks (21). In our trial, NAC decreased 9.6(6.6) score during 10 weeks, which was statistically significant. However, the change of score was not significant in the placebo group. Our results support the hypothesis that NAC is effective in treating medication resistance OCD symptoms in children and adolescents. It is not clearly known that how NAC decreases the severity of OCD symptoms. Nonetheless, its effect is proposed to be through glutamate (12).

The results of this preliminary explanatory trial support that NAC may add to the effect of citalopram in improving some aspects of resistance/control and insight in patients with OCD.

Moreover, the results of this study revealed that NAC plus citalopram improved the different domains of quality of life of patients with OCD. Physical functioning, emotional functioning, and social functioning were improved in the NAC group. However, physical functioning did not change in the placebo group. Of course, it needs to be reminded that no difference was detected between the two groups with respect to the effect on quality of life. No pervious published study examined the effect of NAC on quality of life to compare our results with it. 

This trial also investigated the adverse effects of NAC in treating OCD symptoms. NAC was well tolerated. No serious adverse effect was reported, and the rates of different adverse effects were not different between NAC and placebo groups. Many of the adverse effects were rare. 

There were some limitations, which should be considered. The maximum dosage of NAC was administered from week 6. It is possible that longer duration or higher dosages are required to be more effective for treating OCD symptoms. The sample size was relatively low. Larger sample size may provide more reliable results. In addition, it is not clear whether the effect of NAC is stable in the long term or the symptoms would relapse after discontinuation of NAC. Furthermore, the rater in one of the two centers could correctly guess the allocation of patients in the NAC group. Considering these limitations, the current findings cannot guarantee the recommendation of regular administration of NAC as an adjuvant medication for treating OCD symptoms in children and adolescents. The mechanism of NAC effect on OCD should be investigated. Furthermore, future studies may examine the effect of NAC plus other medications for treating OCD. Considering the effect of NAC on resistance/control over compulsions, further studies may examine if NAC improve tics in children and adolescents with tics disorders or anger attacks. 

Despite the limitations, this was the first randomized double- blind placebo- controlled clinical trial that examined the efficacy and safety of NAC for treating children and adolescents. The current results encourage conducting further trials investigating the efficacy and safety of NAC in treating children and adolescents with OCD.

## Limitations

Further trials with larger sample sizes are needed to be conducted. 

## Conclusion

NAC as an adjuvant agent to SSRIs improves resistance/control over compulsion symptoms in children and adolescents with OCD. Moreover, NAC is well tolerated.
